# Current perspectives on KMT2A fusion proteins and menin inhibition in paediatric acute myeloid leukaemia

**DOI:** 10.1111/febs.70460

**Published:** 2026-02-18

**Authors:** Lydia Elaine Roets, Graeme Greenfield, Katrina Mairead Lappin

**Affiliations:** ^1^ Johnston Cancer Research Centre, Queen's University Belfast UK

**Keywords:** acute myeloid leukaemia, childhood AML, KMT2A fusion proteins, KMT2A rearrangements, paediatric AML

## Abstract

The therapeutic landscape of acute myeloid leukaemia (AML) has evolved beyond the classic ‘7 + 3’/DA regimen, through the approval and incorporation of targeted treatments in both front‐line and relapsed/refractory settings. Indeed, the use of selective BCL‐2 antagonists (e.g. venetoclax) and FLT3 inhibitors (e.g. midostaurin, gilteritinib) which target specific molecular characteristics of leukaemic cells, has enhanced outcomes and survival rates. Arguably one of the most exciting advancements has been the clinical development of menin inhibitors for the treatment of patients harbouring specific genetic aberrations. These abnormalities include rearrangements of the lysine methyltransferase 2A (*KMT2A*) gene, and they occur in approximately one fifth of childhood/paediatric (i.e. infant, adolescent and young adult) AML patients. Spurred on by the recent FDA approval of revumenib, menin inhibitors hold the potential to further shift the treatment paradigm for this patient population. Here, we aim to provide a comprehensive overview of the pathogenesis of *KMT2A* rearrangements, with a focus on KMT2A fusion genes and proteins within paediatric AML patients. Additionally, we summarise the challenges arising from resistance to menin inhibitors, and we touch on the potential of combination therapies to expand the efficacy of menin inhibition and mitigate some of the resistance mechanisms employed by leukaemic clones.

## Graphical abstract

Genetic rearrangements resulting in the expression of KMT2A fusion alleles can lead to dramatic transcriptional disturbances that contribute to the onset of acute leukaemias. Fortunately, menin inhibition has emerged as a promising new class of targeted therapy. These compounds work by blocking the protein–protein interaction between menin and KMT2A, which inhibits the transcription of downstream target genes and limits the progression of KMT2A‐rearranged acute leukaemias.

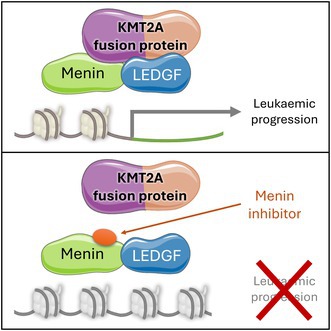

**Abbreviations**
“7 + 3” / DA regimen: daunorubicin and cytarabine standard‐of‐care chemotherapy combination
AL: acute leukaemia
ALL: acute lymphoid leukaemia
AML: acute myeloid leukaemia
BCL‐2: B‐Cell Lymphoma‐2
BMI1: B‐lymphoma Mo‐MLV Insertion region 1
CLL: chronic lymphoid leukaemia
CR: complete remission
CRc: composite complete remission defined as CR or CR with partial or incomplete haematological recovery
CRh: complete remission with partial haematological recovery
CtBP: C‐terminal Binding Protein
CYP33: Cyclophilin 33
DLBCL: diffuse large B‐cell lymphoma
FLA: fludarabine and cytarabine
FLAG/IDA: fludarabine, cytarabine, granulocyte‐colony stimulating factor, and idarubicin
FLT3: FMS‐Like Tyrosine kinase 3
FLT3‐m: mutant FMS‐Like Tyrosine kinase 3
FYRN: FY‐rich N‐terminal
FYRC: FY‐rich C‐terminal
GO: gemtuzumab ozogamicin
H3K27me3: histone H3 lysine 27 tri‐methylation
H3K4me3: histone H3 lysine 4 tri‐methylation
H3K79: histone H3 lysine 79
HCVD: hyper‐cyclophosphamide, vincristine and dacarbazine chemotherapy
HCT: haematopoietic cell transplant
HDAC: Histone deacetylase
HOX: Homeobox
HPC2: Histone Promoter Control 2
HUGO: Human Genome Organisation
KMT2A(r): lysine methyltransferase 2A (rearranged/rearrangements)
KMT2C: lysine methyltransferase 2C
LDAC: low‐dose cytarabine
LEDGF: Lens Epithelium‐Derived Growth Factor
MBD: Methyl‐Binding Domain
MLL1: Mixed Lineage Leukaemia protein 1
MM: multiple myeloma
MPAL: Mixed Phenotype Acute Leukaemia
MEN1: Multiple Endocrine Neoplasia, type 1
MEIS1: Myeloid Ecotropic viral Integration Site 1 homologue
ND: newly diagnosed
NPM1: nucleophosmin 1
NPM1‐m: mutant nucleophosmin 1
NUP214(r): Nuclear Pore complex 214 (rearrangements)
NUP98(r): Nuclear Pore complex 98 (rearrangements)
ORR: overall response rate
PAF(1c): Polymerase‐Associated Factor (1 complex)
PHD: Plant Homeodomain
pts: patients
R/R: relapsed/refractory
SET: Su(var)3–9, Enhancer‐of‐zeste, and Trithorax
SEC: Super Elongation Complex
TARGET: Therapeutically Applicable Research to Generate Effective Treatments
UBTF(‐TD): Upstream Binding Transcription Factor (tandem duplications)
VEN/AZA: venetoclax/azacytidine

## Introduction

Acute myeloid leukaemia (AML) is a molecularly heterogeneous cancer of the bone marrow and peripheral blood that is characterised by the (oligo−/multi‐)clonal proliferation of undifferentiated myeloid blasts or progenitor cells [1, 2]. It tends to be viewed as a disease of the elderly; however, it can develop in children, sometimes as early as a few days after birth [3] or even *in utero*, as translocations have been observed on Newborn Screen/Guthrie test blood spots [4]. Despite some advances in therapeutic options, AML remains difficult to treat. This is particularly the case in very young patients with increased susceptibility to therapy‐related toxicity [5]. Fortunately, collaborative efforts are already in place to better understand the biology of several high‐risk or hard‐to‐treat paediatric cancers. One of which is the Therapeutically Applicable Research to Generate Effective Treatments (TARGET) initiative. A landmark study from their AML project involved genomic analyses of nearly 1000 children and young adults with AML and revealed that the disease differs greatly between younger and older patients, at the genetic level. Specifically, somatic mutations are much more common in older adults with AML, whereas structural alterations are more abundant in younger patients with the disease [6]. A substantial proportion of the structural alterations are due to translocations, where sections of chromosomes shift, and can result in the production of fusion genes and fusion proteins. Indeed, rearrangements involving chromosome band 11q23 and *KMT2A* (*KMT2A*r) are key drivers of acute leukaemias—representing about 15% of paediatric AML cases [7]—and a defining genetic abnormality used by the World Health Organization to help classify AML [8]. These patients often present with high‐risk clinical characteristics, including elevated white blood cell counts and extramedullary involvement [9].

NB: Human Genome Organisation (HUGO) nomenclature (https://www.genenames.org/) is utilised throughout the text, with some genes along with their former synonyms listed here for convenience: *KMT2A* = former *MLL*, *AFF1* = former *AF4*, *MLLT1* = former *ENL*, *MLLT3* = former *AF9*, *AFDN* = former *AF6*, *MLLT10* = former *AF10*, *MLLT11* = former *AF1Q*.

## KMT2A structure and function

**Fig. 1:**
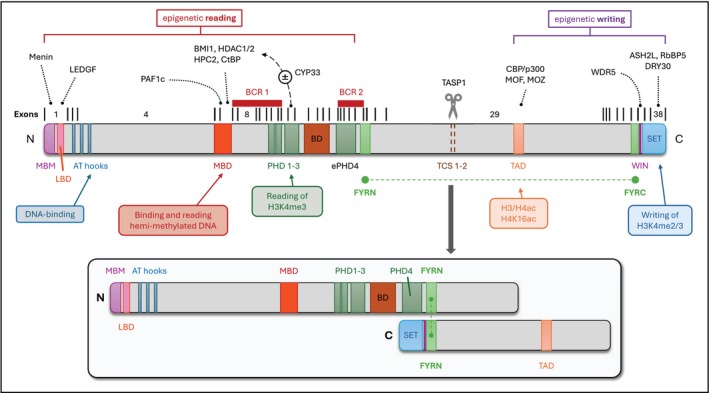
Schematic overview of the structure and function of KMT2A. A full length KMT2A protein is depicted with its functional domains and known interacting proteins. The exon structure and breakpoint cluster regions (BCRs)—based on [17]—are shown above. Following Taspase1‐mediated cleavage at its taspase cleavage sites (TCS), the two KMT2A fragments assemble into a holo‐complex via their FY‐rich N (FYRN) and C (FYRC) terminal domains (shown below). Subsequent binding to target promoter regions occurs via recruitment of menin and LEDGF to the menin‐binding motif (MBM) and LEDGF‐binding domain (LBD), respectively. The methyl‐binding domain (MBD) binds and reads unmethylated or hemi‐methylated DNA. The (extended) plant homeodomains (PHDs) can read the epigenetic markers on histone core particles with the help of the non‐functional bromodomain (BD), while the Su(var)3–9, Enhancer‐of‐zeste and Trithorax (SET) domain allows for epigenetic writing, i.e., di‐ and tri‐methylation of the lysine 4 residue within histone H3 (H3K4me2/3). WD‐40 repeat protein 5 (WDR5) is recruited via the WDR5‐interacting (WIN) domain to enhance KMT2A's reprogramming of the epigenome. Proteins associated with the transactivation domain (TAD) (e.g., CBP/p300, MOF and MOZ) can acetylate nucleosomes to regulate chromatin functional states. CYP33 enables docking of a polycomb repressive complex composed of BMI1, HPC2, CtBP and several HDACs which remove acetyl groups from nucleosomes or transcription factors to reduce gene transcription.

**Fig. 2:**
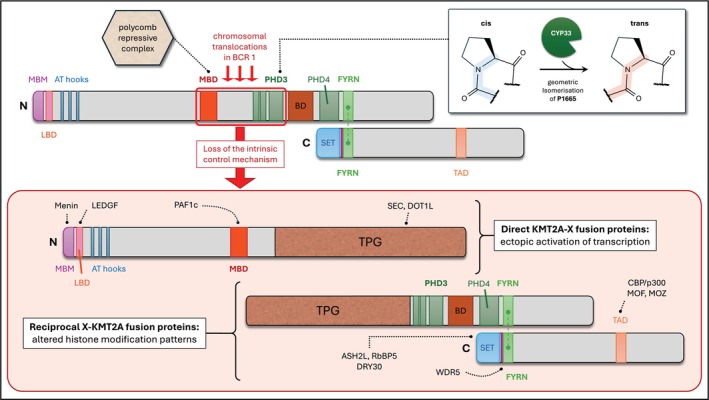
Loss of the intrinsic control mechanism of KMT2A which requires both the presence of both the methyl‐binding domain (MBD) and plant homeodomain 3 (PHD3). PHD3 contains a proline residue at position 1665 which undergoes CYP33‐mediated geometric isomerisation, enabling the subsequent recruitment of a polycomb repressor complex (consisting of BMI1, HPC2, CtBP and HDAC1/2) to the MBD. Chromosomal translocations in AML patients occur within the major breakpoint cluster region (BCR1), effectively destroying the intrinsic regulatory mechanism of KMT2A due to separation of its MBD and PHD3. The disrupted KMT2A portions can be fused to sequences deriving from several different partners (*n* = 112). The N‐terminal portion of KMT2A retains the ability to interact with menin and LEDGF – via its menin‐binding motif (MBM) and LEDGF‐binding domain (LBD), respectively – and can therefore bind to target promoter regions. Depending on the fusion sequence (i.e. whether *AFF1*, *MLLT1*, *MLLT3*, or *MLLT10* are involved), direct KMT2A‐X fusion proteins may recruit the endogenous AFF1 protein complex that contains P‐TEFb and the histone methyltransferases: DOT1L, NSD1 and CARM1. This boosts transcription and results in enhanced epigenetic signatures, that is di‐ and tri‐methylation of lysine 79 with histone H3 (H3K79me2/3). The C‐terminal portion retains its Su(var)3–9, Enhancer‐of‐zeste and Trithorax (SET) domain, as well as binding capacities via the transactivation domain (TAD) for CBP/p300, MOF and MOZ. In some reciprocal *AFF1‐KMT2A* fusion cases, the N‐terminal fused protein sequences are capable of binding both P‐TEFb and the largest subunit of RNA polymerase II to enhance transcriptional elongation. Additionally, DOT1L and NSD1 can still be bound, producing aberrant (promoter‐like) histone signatures (i.e. H3K79me2/3, H3K36me2 and H3K4me2/3) in transcribed gene bodies, that may help reactivate neighbouring genes over time.

*KMT2A*, or *MLL1* as it was previously known, encodes a histone H3 lysine 4 methyltransferase which plays a crucial role in regulating gene expression during embryonal development and the maintenance of haematopoietic stem cells [10]. The KMT2A protein is processed by the endopeptidase, Taspase1, into two amino (N320) and carboxyl (C180) terminal fragments [11], which reassociate (via their FYRN and FYRC domains) to form a molecular hub for the assembly of larger multiprotein complexes in the nucleus (Fig. 1). Simply put, the N‐terminal portion of KMT2A is responsible for promoter binding and reading of chromatin signatures, while the C‐terminal portion—which contains a Su(var)3–9, Enhancer‐of‐zeste and Trithorax (SET) domain—methylates lysine 4 within histone H3 to promote the transcription of target genes, including *HOX* genes [12, 13] (Fig. 1). It is worth noting that transcriptional profiling of isogenic wildtype and KMT2A knockout murine fibroblasts revealed that more genes were upregulated than downregulated in the knockout situation, suggesting the presence of a molecular switch that toggles between transcriptional activation and repression [14]. We now know that this is due to a conformational change following CYP33—a prolyl‐peptidyl isomerase—binding to the enhanced plant homeodomain 3 (ePHD3) subdomain within KMT2A. Typically, ePHD3 binds to di‐ and tri‐methylated lysine‐4 residues within histone H3 [15]. However, CYP33‐mediated *cis‐trans* isomerisation of the proline‐1665 residue in KMT2A prevents this and instead enables the subsequent recruitment of a polycomb repressor complex (i.e. BMI1, HPC2, CtBP and HDAC1/2) to the methyl‐DNA binding domain (MBD) of KMT2A [16] (Fig. 2). The distinct domains within KMT2A which we are still learning more about [17], along with the various proteins and transcription factors that contribute to the functional KMT2A multiprotein complex, are crucial for regulating target genes during embryonic haematopoietic stem cell development [18] and adult haematopoiesis [10].

Given the subtle but fundamental roles of wildtype KMT2A in haematopoiesis, it is no surprise that genetic rearrangements resulting in the expression of KMT2A fusion alleles can lead to dramatic transcriptional disturbances that contribute to the onset of acute leukaemias. Indeed, aberrant H3K79 methylation profiles [19] and upregulated *HOXA*/*MEIS1* gene signatures [20] are hallmark features and well‐established pathomechanisms of KMT2Ar acute leukaemias. While KMT2A rearrangements affect the vast majority (≥ 70%) of infant acute lymphoblastic leukaemia (ALL) cases and a substantial proportion of adult cases, KMT2Ar ALL is beyond the scope of this article, and interested readers are instead directed to [21, 22, 23] for further information.

Chromosomal translocations involving human *KMT2A* (NM_001412597.1) and 112 partner genes have been identified so far [17]. However, the majority of recurrent *KMT2A*r leukaemic translocations involve relatively few fusion partner genes. For instance, ~ 80.7% of cases in a paediatric cohort of 1256 patients with KMT2Ar AML were characterised by six fusion genes, including *KMT2A::MLLT3*/*t*(9;11) (p22; q23) (43.3%), *KMT2A::MLLT10*/*t*(10;11) (p12; q23) (17.4%), *KMT2A::AFDN*/*t*(6;11) (q27; q23) (7.3%), *KMT2A::ELL*/*t*(11; 19) (q23; p13.1) (6.0%), *KMT2A::MLLT1*/*t*(11; 19) (q23; p13.3) (4.5%) and *KMT2A::MLLT11 t*(1; 11) (q21; q23) (2.2%) [24]. Interestingly, four of these partner proteins (i.e. MLLT3/AF9, MLLT10/AF10, ELL and MLLT1/ENL) are involved in transcriptional elongation, as they either bind directly or indirectly—via Super Elongation Complexes (SEC)—to RNA polymerase II [25, 26, 27, 28].

With such a comprehensive list of KMT2A fusion partners, two key concepts emerge—what are the molecular consequences of KMT2A translocations and how important are the contributions of the different fusion partners to the underlying cancer biology of KMT2Ar AML?

With regards to the first point, *KMT2A* rearrangements usually result in physical separation of the MBD from downstream plant homeodomain (PHD) regions, resulting in loss of the intrinsic control mechanism of the KMT2A protein and separating its epigenetic reading and writing functions. Consequently, both separated portions of KMT2A become constitutively active, regardless of their fused protein sequences. The direct KMT2A‐X fusions still bind via menin/LEDGF and the PAF complex to chromatin but are incapable of transcriptional repression as they lack the necessary PHDs (Fig. 2). This was effectively demonstrated by the artificial fusion of PHD to existing KMT2A‐X proteins, which then enabled the subsequent recruitment of the polycomb repressor complex and eliminated the oncogenic properties of the KMT2A‐X fusion proteins [29, 30]. Reciprocal X‐KMT2A fusion proteins which retain their ePHD3 chromatin reader and SET domain are unable to bind the polycomb repressor complex, regardless of CYP33 binding, due to loss of the MBD (Fig. 2). This is not dissimilar to other chromosomal translocations, such as the oncogenic BCR‐ABL1 translocation in chronic myeloid leukaemia which destroys an intrinsic control mechanism within the ABL kinase, producing a constitutively active BCR‐ABL1 fusion protein [31].

Delving beyond the physical separation of the MBD and PHD, the *KMT2A* gene contains a major breakpoint cluster region (BCR1) between intron 7 and exon 13, and a minor BCR (BCR2) located further downstream between exons 21 and 25. Interestingly, while breakpoints in ALL patients are found in both regions, AML breakpoints seem to exclusively occur in BCR1 [32]. The focus on *KMT2A* breakpoint distribution stems from correlations with the outcome of KMT2Ar leukaemia patients. More specifically, breakpoints within exon 11 or intron 11 of *KMT2A* are typically associated with poorer clinical outcomes [33]. This is because these breakpoints can alter or destroy the structure of the cysteine‐histidine‐rich PHD1‐3 domain thereby compromising its associated functions, which can: (a) limit functional dimerisation [34]; (b) enhance the stability of KMT2A fusion proteins by interfering with the binding of CDC34 (to PHD2) [35] or ECS^ASB2^ [36], thereby rendering them resistant to proteasomal degradation; and (c) impair CYP33 binding leading to loss of the transcriptional repressor activity of KMT2A [37]. While AML patient breakpoints exhibit a preferential KMT2A intron 9 breakage which decreases slightly with age, further analysis of breakpoint localisation within subregions of BCR1 (i.e. exon9‐intron10 and exon11‐exon13), revealed that certain fusion genes have selective breakpoint preferences (which probably has something to do with the respective functions of the fusion partners) and that these breakpoint tendencies also seemed to change with age [32]. For example, *KMT2A::AFF1* and *KMT2A::ELL* patients exhibit opposite predispositions, that is infant *KMT2A::AFF1* patient breakpoints predominantly localise to intron 11, while adult *KMT2A::AFF1* patient breakpoints shift to introns 9 and 10; whereas infant *KMT2A::ELL* patients have a preference for intron 9 breakpoints, while paediatric and adult *KMT2A::ELL* patient breakpoints shift towards intron 11 [32].

Analysis of large cohorts of patients with integrated molecular data suggests that KMT2A fusion partners carry prognostic significance. For instance, *KMT2A::MLLT3* and *KMT2A::MLLT11* tend to be associated with more favourable survival outcomes in children, whereas *KMT2A::MLLT10* and *KMT2A::AFDN* are among the KMT2A rearrangements with the poorest prognoses in children [38]. Furthermore, *KMT2A::MLLT3* is categorised as intermediate risk according to the 2022 edition of the European Leukaemia Net recommendations, with other KMT2Ar associated with less favourable outlooks [39]. Although it should be noted that the prognosis of children with *KMT2A::MLLT3* can be adversely affected by the presence of secondary chromosome aberrations [24, 40] and/or by a marrow morphology that is different from acute monoblastic leukaemia (FAB‐M5) [38]. In a similar vein, more recent evidence suggests that the aforementioned favourable risk stratification of *KMT2A::MLLT11* should be revised to intermediate risk [24]. A retrospective international study of 1130 paediatric KMT2Ar AML patients categorised cases with *KMT2A::MLLT10*, *KMT2A::AFDN*, *KMT2A::MLLT1*, *KMT2A::AFF1*/*t*(4; 11) (q21; q23), and *KMT2A::ABI1*/*t*(10; 11) (p11.2; q23) rearrangements as high risk, with remaining cases allocated to a non‐high risk group [9]. The high‐risk cohort exhibited a higher cumulative incidence of relapse (59.7% for high risk vs 35.2% for non‐high risk; *P* < 0.0001), lower event‐free survival (EFS) rates (30.3% vs 54.0%; *P* < 0.0001), and worse overall survival (OS) (49.2% vs 70.5%; *P* < 0.0001) [9].

The remaining list of recurrently diagnosed translocation partner genes is long, but these cases are rare and not of pressing clinical relevance. However, they should not be overlooked and a systemic classification about their functions has already been proposed [41]. The presence of reciprocal X‐KMT2A fusion proteins is yet another important factor, as demonstrated by the chromatin opening capability of *AFDN::KMT2A* and its ~ 12‐fold enhancement of the KMT2Ar transcriptome [42]. Gene dysregulation of this magnitude likely contributes to the onset of pre‐leukaemic clones that then undergo selection to overt leukaemic cells. However, the enhanced transcriptome could also result in the expression of more druggable target proteins that may sensitise the leukaemic cells, ultimately translating to better clinical outcomes. It is possible that leukaemic cells lacking a reciprocal fusion protein and therefore possessing a more restricted transcriptome, might be more chemo‐resistant. As such, the identification of reciprocal fusion proteins could provide important prognostic information and impact disease monitoring.

## Current and future therapies involving menin inhibition

Children with KMT2Ar AML are associated with an intermediate 5‐year event‐free survival rate of ~ 45% and an overall survival rate of ~ 63%, highlighting an unmet clinical need for this patient population [9]. At present, strategies to treat KMT2Ar leukaemias are based on canonical risk stratification [39], although menin inhibition has emerged as a promising new class of targeted therapy for KMT2Ar AML [2].

### Biological basis for menin inhibition

Menin is a chromatin adaptor protein, encoded by the *MEN1* gene, that participates in epigenetic gene regulation through its interactions with various proteins—including chromatin modifying proteins and transcription factors [43]. It has paradoxical roles, acting as both a tumour suppressor and an oncogenic co‐factor, depending on the cellular context. Mutations in the *MEN1* gene that disrupt its functionality are typically associated with Multiple Endocrine Neoplasia Type 1 (MEN1) syndrome [44, 45], highlighting the tumour suppressive activity of the gene in neuroendocrine tissues. In contrast, menin serves as an oncogenic co‐factor in haematopoietic pathological states [46]. Indeed, menin was initially identified as an essential oncogenic co‐factor for KMT2Ar leukemogenesis in a mouse model back in 2005 [47]. Since then, there has been considerable development into several iterations of highly selective small molecule menin inhibitors (summarised in Table 1), with eight compounds currently at various clinical stages for the treatment of AML: ziftomenib (KO‐539), revumenib (SNDX‐5613), bleximenib (JNJ‐75276617), BN104, icovamenib (BMF‐219), DS‐1549, enzomenib (DSP‐5336) and balamenib (ZE63‐0302) (summarised in Table 2). The MEN1 inhibitor (or MI) class of drugs (listed in Table 1) has culminated in the commercially available ziftomenib, whereas the preclinical precursor VTP‐50469 has led to revumenib which is currently the only FDA‐approved menin inhibitor for the treatment of relapsed/refractory (R/R) AML [48]. With a generally favourable safety profile in patients, most of the adverse effects associated with these agents are manageable and reversible through dose adjustments and supportive care [2].

**Table 1:** Overview of the menin inhibitors and their precursors developed (as of June 2025).

Menin inhibitor	Inhibitor class	Sponsor	References
MI‐2	MI‐derived	Kura Oncology	[74]
MI‐3			[74]
MI‐2‐2			[49]
MIV‐6 (probe)			[75]
MI‐463			[50]
MI‐503			[50]
MI‐538			[76]
MI‐1481			[77]
MI‐3454			[78]
Ziftomenib (KO‐539)			[79]
VTP‐50469	VTP/SNDX	Syndax Pharmaceuticals	[80]
Revumenib (SNDX‐5613)			[81]
Bleximenib (JNJ‐75276617)	Other	Janssen Research & Development	[82]
Enzomenib (DSP‐5336)	Other	Sumitomo Pharma	[83]
DS‐1594	Other	Daiichi Sankyo	[84]
BN104	Other	BioNova Pharmaceuticals	[85]
BMF‐219	Covalent	Biomea Fusion Inc.	[86]
Balamenib (ZE63‐0302)	Other	Eilean Therapeutics	
BAY‐155 (probe)	Other	Bayer AG	[87]
HMPL‐506	Other	HUTCHMED	[88]

**Table 2:** Summary of the clinical data involving menin inhibition as monotherapies or in combination with standard‐of‐care and/or more targeted therapies. *Efficacy data is in relation to the KMT2Ar AML patient population (i.e., the data for ‘Participant population’ cohorts in brackets has been excluded) where possible. Trials in bold indicate those with paediatric elements. “7 + 3” = cytarabine + daunorubicin standard‐of‐care therapy, AL = acute leukaemia, AML = acute myeloid leukaemia, CLL = chronic lymphoid leukaemia, CR = complete remission, CRc = composite complete remission defined as CR or CR with partial or incomplete haematological recovery, CRh = complete remission with partial haematological recovery, DLBCL = diffuse large B‐cell lymphoma, FLA = fludarabine and cytarabine, FLAG/IDA = fludarabine, cytarabine, granulocyte‐colony stimulating factor and idarubicin, FLT3‐m = mutant FMS‐Like Tyrosine kinase 3, GO = gemtuzumab ozogamicin, HCT = haematopoietic cell transplant, HCVD = hyper‐cyclophosphamide + vincristine + dacarbazine chemotherapy, KMT2Ar = KMT2A rearranged/rearrangements, LDAC = low‐dose cytarabine, MM = multiple myeloma, ND = newly diagnosed, NPM1‐m = mutant nucleophosmin 1, NUP214r = nucleoporin 214 rearrangements, NUP98r = nucleoporin 98 rearrangements, ORR = overall response rate, pts = participants, R/R = relapsed/refractory, RP2D = Recommended phase II doseUBTF‐TD = upstream binding transcription factor tandem duplication, VEN/AZA = venetoclax/azacytidine.

Menin inhibitor	Clinical trial	Phase	Treatment	Participant population	Age eligibility	Location	Status	Efficacy*	Adverse events	Refs
Ziftomenib (KO‐539)	KOMET‐001 (NCT04067336)	I/II	Monotherapy	R/R AML (Phase Ia) with KMT2Ar & NPM1‐m subtypes (Phase Ib); ND & R/R NPM1‐m AML (Phase II)	> 18 years	Multi‐site: US and Europe	Phase 1 completed; Phase 2 ongoing	CR/CRh = 25% (9/36pts) at 600 mg	Differentiation syndrome & tumour lysis syndrome (particularly in KMT2Ar pts)	[89]
	KOMET‐007 (NCT05735184)	I	Combined with “7 + 3”	ND (+ R/R ongoing) KMT2Ar (or NPM1‐m) AML	> 18 years	Multi‐site: US and Europe	Recruiting (data cut‐off June 2024)	CRc = 90% (9/10pts) and MRD‐ = 83% (5/6pts) at 200 mg; CRc = 63% (5/8pts) and MRD‐ = 100% (3/3pts) at 400 mg	Neutropenia; Thrombocytopenia; Anaemia	[90]
			Combined with VEN/AZA	R/R AML with KMT2Ar (or NPM1‐m)	> 18 years	Multi‐site: US and Europe	Recruiting (data cut‐off June 2024)	ORR = 43% (3/7pts) and CRc = 29% (2/7pts) at 200 mg; ORR = 33% (2/6pts) and CRc = 17% (1/6pts) at 400 mg	Differentiation Syndrome; Cytopenias; Pneumonia	[91]
	KOMET‐008 (NCT06001788)	I	Combined with FLAG/IDA or LDAC	R/R AML with KMT2Ar (or NPM1‐m)	> 18 years	Multi‐site: US and Europe	Recruiting			[92]
	**ITCC‐101/APAL2020K (NCT06376162)**	I	Combined with FLA	R/R AML with KMT2Ar (or NPM1‐m or NUP‐98r)	0–21 years	Multi‐site: US and Europe	Recruiting			[93]
	NCT06930352	II	Monotherapy	KMT2Ar or NPM1‐m AML ineligible for standard therapy	≥ 18 years	US	Recruiting			[94]
	**NCT06448013**	I	Combined with venetoclax and GO	R/R AML or MPAL with KMT2Ar, NPM1‐m, NUP98r, UBTF‐TD, or HOX pathway mutation	≥ 3–21 years	US	Recruiting			[95]
Revumenib (SNDX‐5613)	AUGMENT 101 (NCT04065399)	I	Monotherapy	Phase I: R/R KMT2Ar + NPM1‐m AL	≥ 30 days	Multi‐site: US	Completed	ORR = 53% (32/60pts); CR/CRh = 30% (18/60pts) and MRD‐ in 14/18pts	Differentiation syndrome; QT prolongation; Neutropenia	[81]
		II		Phase II: R/R KMT2Ar AL (& NPM1‐m AML)	≥ 30 days	Multi‐site: US and Europe	KMT2Ar cohort: Completed; NPM1‐m cohort: Ongoing	ORR = 63.2% (36/57pts); CR/CRh = 22.8% (13/57pts) and MRD‐ in 7/10pts		[96]
	**AUGMENT 102 (NCT05326516)**	I	Combined with FLA	R/R AL with KMT2Ar, NPM1‐m, or NUP98r	≥ 30 days	Multi‐site: US and Europe	Completed	CRc = 44% (4/9pts) at dose level 1; CRc = 50% (9/18pts) at dose level 2; 92% MRD‐ (12/13pts)	Thrombocytopenia; Anaemia	[97]
	NCT05886049	I	Combined with daunorubicin & cytarabine	ND AML with KMT2Ar or concurrent NPM1‐m & FLT3‐m	18–75 years	Multi‐site: US	Recruiting			[98]
	**RAVAML (NCT06177067)**	I	Combined with VEN/AZA	R/R AML or ALAL with KMT2Ar, NPM1‐m, NUP98, etc.	1–30 years	Multi‐site: US	Recruiting			[99]
	SAVE (NCT05360160)	I/II	Combined with ASTX727 + venetoclax	R/R AML or MPAL with KMT2Ar, NPM1‐m, or NUP98r; ND AML ineligible for intensive chemotherapy	≥ 12 years	Multi‐site: US	R/R: Completed; ND: Recruiting (data cut‐off July 2024)	ORR = 88% (23/26pts); 74% MRD‐ (17/23pts); CR/CRh = 58% (15pts); 93% MRD‐ (13/14pts)	QT prolongation; Neutropenia; Hyper‐phosphataemia; Nausea	[100]
	**NCT06575296**	I	Post‐transplant maintenance after allogeneic HCT	AL with KMT2Ar or NPM1‐m	≥ 2 years	US	Recruiting			[101]
	Beat AML (NCT03013998)	Ib	Combined with VEN/AZA	ND AML with KMT2Ar or NPM1‐m	≥ 60 years	Multi‐site: US	Recruiting	ORR = 100% (9/9pts); CRc = 89% (8/9pts)		[102]
	NCT06222580	I	Combined with gilteritinib	R/R FLT3‐m AML with KMT2Ar (or NPM1‐m)	≥ 18 years	Multi‐site: US	Recruiting			[103]
Bleximenib (JNJ‐75276617)	**cAMeLot‐1 (NCT04811560)**	I/II	Monotherapy	R/R AL with KMT2Ar, NPM1‐m, NUP98r, or NUP214r (Phase I); R/R AML with KMT2Ar or NPM1‐m (Phase II)	≥ 2 years (paediatric cohort) & ≥ 18 years (Phase I); ≥ 18 years (Phase II)	Multi‐site: North & South America, Asia, Australia, Europe	Recruiting (data cut‐off April 2023)	ORR = 50% (n = 4) at highest dose level in Phase I	Differentiation syndrome; Neutropenia; Thrombocytopenia	[104]
	**cAMeLot‐2** **(NCT06852222)**	III	Combined with VEN/AZA	ND AML with KMT2Ar or NPM1‐m ineligible for intensive chemotherapy	≥ 18 years	Multi‐site: North & South America, Asia, Australia, Europe	Recruiting			[105]
	**ALE1002 (NCT05453903)**	Ib	Combined with intensive cytarabine + daunorubicin/idarubicin	ND (& R/R) AML with KMT2Ar or NPM1‐m (& other menin‐sensitising genetics)	≥ 12 years (≥ 12–18 years for R/R cohort)	Multi‐site: North America, Australia, Europe	Recruiting (data cut‐off July 2024)	ORR = 88% (7/8pts); CR/CRh = 88% (7/8pts)	Neutropenia; Thrombocytopenia; Anaemia	[106]
			Combined with VEN/AZA	ND & R/R AML with KMT2Ar or NPM1‐m			Recruiting (data cut‐off February 2025)	R/R efficacy: 50 mg (26pts): ORR = 76%; CRc = 32%; 100 mg (24pts): ORR = 79%; CRc = 54%; RP2D = 100 mg twice daily; No *MEN1* resistance mutations detected	Neutropenia; Thrombocytopenia; Anaemia	[107]
Enzomenib (DSP‐5336)	NCT04988555	I/II	Monotherapy	R/R AL with KMT2Ar (or NPM1‐m or other menin‐sensitising genetics)	≥ 18 years	Multi‐site: US and Japan	Recruiting (data cut‐off June 2024)	ORR = 59.1% (13/22pts); CR + CRh = 22.7% (5/22pts) at the right dose between 140 and 300 mg	Nausea; Vomiting; Pneumonia; Sepsis	[108]
DS‐1594	NCT04752163	I/II	Combined with VEN/AZA or mini‐HCVD	Phase I: R/R AL; Phase II: R/R AL with KMT2Ar or NPM1‐m	≥ 18 years	MD Anderson Cancer Center	Completed	Awaiting results		[109]
BN104	NCT06052813	I/II	Monotherapy	R/R AML with KMT2Ar and/or NPM1‐m	≥ 18 years	China	Recruiting (data cut‐off June 2024)	ORR = 88.9% (8/9pts); CR/CRh = 33.3% (3/9pts)	Neutropenia; Pneumonia; QT prolongation; Differentiation syndrome	[85]
	NCT06746519	I/II	Combined with “7 + 3” or VEN/AZA	ND & R/R AML with KMT2Ar, NPM1‐m, or NUP98r	≥ 18 years	China	Recruiting			[110]
BMF‐219	COVALENT‐101 (NCT05153330)	I	Monotherapy	Cohort 1: R/R AL (cohorts 2–4 include pts with DLBCL, MM, & CLL)	≥ 18 years	Multi‐site: US and Europe	Terminated (data cut‐off July 2023)	CR: 2/5 evaluable pts	Differentiation syndrome; Vomiting	[86]
Balamenib (ZE63‐0302)	NCT06780124	I	Monotherapy	Healthy volunteers	≥ 18–55 years	Australia	Recruiting			[111]

These compounds work by specifically targeting a central hydrophobic pocket on menin's surface to prevent the protein–protein interaction between menin and KMT2A (i.e., either the N‐terminal portion of wildtype KMT2A or oncogenic fused versions of the protein) [49]. This effectively inhibits KMT2A‐dependent transcription of downstream target genes, thereby blocking an important component in the pathogenesis of KMT2Ar AML. Importantly, the first two menin inhibitors taken forward for *in vivo* work—MI‐463 and MI‐503—blocked the progression of KMT2Ar AML in mice, without impairing normal haematopoiesis [50]. Furthermore, another study found that treatment of KMT2Ar AML cell lines with the same two menin inhibitors led to increased ubiquitylation and reduced protein stability of menin, suggesting an additional aspect of menin inhibition beyond physical disruption of the menin‐KMT2A interaction [51]. Leaning into this theme, recent work has demonstrated altered gene expression following menin inhibition, beyond the typical silencing of the *HOX*‐ and *MEIS1*‐dependent oncogenic transcriptional programme. For instance, menin loss (via genetic knockout and VTP‐50469 treatment) resulted in redistribution of KMT2A from (menin‐dependent) active genes to a subset of silent bivalent genes (i.e. genes which are concurrently marked by activating H3K4me3 and repressive H3K27me3 modifications to maintain a transcriptionally inert state, poised for activation or stable repression), which elevated menin‐independent KMT2A gene expression [52]. Moreover, these upregulated genes included major histocompatibility class I (MHC‐I) molecules, suggesting that the efficacy of menin inhibition may involve the loss of immune evasion in KMT2Ar leukaemic blasts [52]. Additional supporting evidence comes from a recent study demonstrating bleximenib‐induced (*MEIS1*‐independent) upregulation of HLA class I and II expression in leukaemic blasts, along with enhanced T‐cell mediated cytotoxicity [53].

In summary, the anti‐leukaemic effects of menin inhibitors can be achieved through both canonical and non‐canonical mechanisms, via disruption of leukaemogenic transcriptional programs while also reshaping the immunogenic landscape of KMT2Ar AML. As such, menin inhibition remains a promising therapeutic strategy with potential to directly suppress leukaemic proliferation and enhance immune‐mediated clearance of leukaemic cells.

### Challenges and resistance mechanisms

Menin inhibitors have great potential for addressing unmet needs in various subtypes of genetically defined acute leukaemias, typically characterised by (menin‐dependent) upregulation of *HOX* gene expression [54]. However, enrolling paediatric patients in clinical trials is challenging, mainly due to the small number of affected individuals. The annual incidence of newly diagnosed paediatric AML cases globally is fewer than 1000, with an estimated annual incidence of around 350 cases of newly diagnosed and recurrent KMT2Ar AML among paediatric patients [55]. Without global collaboration, these limitations can impede insights into side effects and efficacy across KMT2A fusion partners.

Drug resistance is a recurring theme in the development of cancer treatments, and menin inhibition is no exception. Two forms of menin inhibitor resistance have been described: *MEN1* mutations and non‐*MEN1* mutation‐driven mechanisms for example through cellular adaptation processes. In the case of the former, somatic *MEN1* mutations were identified in patients that received revumenib (38.7% in the AUGMENT‐101 phase 1 trial), as well as in patient‐derived xenograft models and an unbiased base‐editor screen [56]. Some of these mutations (including M327I, G331D, G331R and T349M) affect residues near the KMT2A binding site in menin, generating steric hindrance with revumenib to effectively attenuate binding of the inhibitor with menin, but without impacting the menin‐KMT2A interaction [57]. The G331D mutation is particularly interesting as it results in a very slow dissociation of KMT2A from menin, suggesting that the menin^G133D^‐KMT2A interaction might be even more challenging to dissociate with small molecule inhibitors, when compared to wildtype and other mutant versions of menin [57].

Menin inhibitor resistance arising in the absence of *MEN1* mutations is much broader, ranging from alterations in parallel or downstream pathways [58, 59] to treatment‐induced clonal evolution despite on‐target efficacy of inhibitors at the transcriptional level [60, 61]. Delving deeper into the altered signalling pathways, loss of non‐canonical polycomb repressive complex 1.1 (PRC1.1)‐mediated signalling has been linked with menin inhibitor resistance, via epigenetic reactivation of non‐canonical menin‐KMT2A targets, such as *MYC* [58]. This is problematic because *MYC* is a critical oncogene that has been implicated in AML pathogenesis [62] and the promotion of cytotoxic drug resistance [63]. Moreover, after establishing that the UTX‐KMT2C/D complex contributes to the efficacy of menin inhibition by inducing a myeloid differentiation program, a patient‐derived xenograft model of AML harbouring a *KMT2C* mutation was found to be resistant to VTP‐50469 [59]. This effectively highlights acquired mutagenesis of essential, non‐driver epigenetic regulators as potential adaptive pathways contributing towards menin inhibitor resistance.

### Strategies to mitigate resistance

Some of the *MEN1* mutations (i.e. M327I and T349M) that confer resistance to revumenib could potentially be addressed with newer iterations of menin inhibitors, such as bleximenib (JNJ‐75276617) [64] and balamenib (ZE63‐0302) [65] which circumvent the acquired steric hindrance through a unique binding mode. With this in mind, it seems prudent to standardise *MEN1* mutation status monitoring during menin inhibition therapy, as this could help guide treatment decisions and ultimately improve patient outcomes.

Combination treatments with menin inhibitors are also being pursued both in preclinical studies (Table 3) and clinical trials (Table 2) to either prevent or overcome intrinsic and acquired mechanisms of resistance.

**Table 3:** Preclinical studies combining menin inhibition with agents targeting epigenetic regulation, DNA damage, the cell cycle, kinase inhibition and apoptosis. CDX = cell line‐derived xenograft, PDX = patient‐derived xenograft.

Menin inhibitor	Co‐treatment agent	KMT2Ar	Resistance mechanism	Model	References
VTP‐50469	Mezigdomide (cereblon E3 ligase modulator) (IKAROS degrader)	KMT2A‐MLLT10	MEN1^T349M^ mutation	PDX murine model of AML	[112]
MI‐503	SD70 (KDM4C inhibitor)	KMT2A‐MLLT3	N/A	MOLM‐13 CDX murine model of AML	[113]
VTP‐50469	OTX015 (pan‐BET protein inhibitor)	KMT2A‐MLLT3	N/A	MOLM‐13 CDX murine model of AML	[114]
Revumenib (SNDX‐5613)	OTX015 (pan‐BET protein inhibitor)	KMT2A‐MLLT3 + FLT3‐TKD	N/A	PDX murine models of AML	[114]
Ziftomenib (KO‐539)	OTX015 (pan‐BET protein inhibitor)	KMT2A‐MLLT3 + FLT3‐TKD	N/A	PDX murine model of AML	[79]
Ziftomenib (KO‐539)	All‐trans‐retinoic acid (ATRA)	KMT2A‐MLLT3 & KMT2A‐AFF1	N/A	MOLM‐13 and MV4‐11 cell lines	[115]
Ziftomenib (KO‐539)	ORY‐1001 (LSD1 inhibitor)	KMT2A‐MLLT3 & KMT2A‐AFF1	N/A	MOLM‐13 and MV4‐11 cell lines	[115]
VTP‐50469	FHD‐286 (BRG1/BRM‐selective ATPase inhibitor)	KMT2A‐MLLT3, KMT2A‐AFF1, KMT2A‐MLLT3 + FLT3‐TKD	N/A for AML cell lines; *KMT2C* mutation in patient cells	MOLM‐13, MV4‐11 & KMT2Ar patient cells	[116]
MI‐2‐2	EPZ004777 (DOT1L inhibitor)	KMT2A‐MLLT3	N/A	Syngeneic murine model of AML	[117]
Revumenib (SNDX‐5613)	GNE‐781 (CBP/p300 inhibitor)	KMT2A‐MLLT3	N/A	MOLM‐13 CDX murine model of AML	[114]
Bleximenib (JNJ‐75276617)	Azacytidine (DNA methyltransferase inhibitor)	KMT2A‐MLLT3	N/A	MOLM‐13 CDX murine model of AML	[64]
Ziftomenib (KO‐539)	Olaparib and talazoparib (PARP inhibitors)	KMT2A‐MLLT3 & KMT2A‐AFF1	N/A	MOLM‐13 and MV4‐11 cell lines	[115]
Ziftomenib (KO‐539)	Mycophenolate mofetil (MMF) (purine biosynthesis inhibitor)	KMT2A‐MLLT3 & KMT2A‐AFDN	N/A	PDX murine model of AML	[118]
Ziftomenib (KO‐539)	GSK3326595 and JNJ‐64619178 (PRMT5 inhibitors)	KMT2A‐MLLT3 & KMT2A‐AFF1	N/A	MOLM‐13 and MV4‐11 cell lines	[115]
VTP‐50469	Palbociclib (CDK4/6 inhibitor)	KMT2A‐MLLT3	KMT2C mutation	PDX murine model of AML	[59]
Ziftomenib (KO‐539)	Ribociclib and palbociclib (CDK4/6 inhibitors)	KMT2A‐MLLT3 & KMT2A‐AFF1	N/A	MOLM‐13 and MV4‐11 cell lines	[115]
VTP‐50469	Selutinib (MEK1/2 inhibitor)	KMT2A‐MLLT10	*KRAS* mutation in patient cells	PDX murine model of AML with mutant *KRAS*	[69]
VTP‐50469	Selutinib (MEK1/2 inhibitor)	KMT2A‐PICALM	*NRAS* mutation in patient cells	PDX murine model of AML with mutant *NRAS*	[69]
Ziftomenib (KO‐539)	MIK‐2206 (AKT inhibitor)	KMT2A‐MLLT3 & KMT2A‐AFF1	N/A	MOLM‐13 and MV4‐11 cell lines	[115]
Ziftomenib (KO‐539)	Gilteritinib (FLT3 inhibitor)	KMT2A‐MLLT3 & KMT2A‐AFF1	N/A	MOLM‐13 and MV4‐11 cell lines	[115]
Ziftomenib (KO‐539)	Venetoclax (BCL‐2 inhibitor)	KMT2A‐MLLT3 + FLT3‐TKD	N/A	PDX murine model of AML	[79]
Revumenib (SNDX‐5613)	Venetoclax (BCL‐2 inhibitor)	KMT2A‐MLLT3	N/A	MOLM‐13 CDX murine model of AML	[119]
VTP‐50469	Venetoclax (BCL‐2 inhibitor)	KMT2A‐AFDN	PRC1.1 loss	Syngeneic murine model of AML	[58]
Bleximenib (JNJ‐75276617)	Venetoclax (BCL‐2 inhibitor)	KMT2A‐MLLT3	N/A	MOLM‐13 CDX murine model of AML	[64]
Bleximenib (JNJ‐75276617)	Azacytidine + Venetoclax	KMT2A‐MLLT3	N/A	MOLM‐13 CDX murine model of AML	[64]
Balamenib (ZE63‐0302)	FLT3 + BCL2 inhibitors	KMT2A‐AFF1	MEN1^M327I^ mutation	MV4‐11 cells; MOLM‐13 CDX murine model of AML	[65]

While KMT2Ar AML typically has a lower incidence of co‐existing gene mutations, a number of tyrosine kinase‐PI3K‐RAS pathway mutations (*FLT3, NRAS*, *KRAS*, *PTPN11* and *BRAF*) have been reported to co‐occur and confer more aggressive, high‐risk leukaemic profiles [66, 67], presumably by promoting the clonal expansion of KMT2Ar leukaemic cells, as demonstrated in a retroviral AML mouse model [68]. Although resistance to menin inhibitors in these unfavourable AML subsets hasn't yet been described *per se*, combination therapies with menin and FLT3 or MEK inhibitors (which target the RAS/MAPK pathway) are already underway in clinical (Table 2) and preclinical [69] (Table 3) settings.

The rationale for combining menin inhibition with broadly effective agents (e.g., chemotherapy, azacitidine, or venetoclax) is based on leveraging distinct mechanisms of action to achieve both cytotoxic debulking and differentiation of leukaemic cells. Interestingly, these combination treatments may even help mitigate the risk of differentiation syndrome [70], which is a commonly occurring drug‐related adverse event associated with menin inhibitor monotherapies (Table 2). While there is a logical basis for combining menin inhibition with current standard‐of‐care approaches, additional preclinical work is required to better understand the mechanistic basis of and optimise any additive/synergistic effects. Further research should explore the optimal sequencing of these double, triple and even quadruple combination treatments (including other targeted therapies i.e. FLT3 and MEK inhibitors): for instance, whether the agents should be given concurrently or sequentially, and whether certain components could be dropped over time. Comparative evaluation of differentiation syndrome rates, myelosuppression, QTc impact, minimum residual disease clearance and response durability will be crucial going forward. These evolving strategies should continue to expand our understanding of other resistance pathways and hopefully pave the way for novel or improved therapeutic approaches to refine treatment paradigms and enhance remission rates.

### Clinical positioning of menin inhibitors

Menin inhibition is one of the latest additions to the growing arsenal of targeted AML treatments, particularly for subsets of patients harbouring *KMT2A* rearrangements or *NPM1* mutations. The precise mechanism underpinning the reliance of this latter AML subtype on the menin‐KMT2A interaction is unclear but is presumably due to a loss of nuclear function (as mutated NPM1 persists in the cytoplasm), culminating in upregulation of *HOX* gene expression which in turn drives leukemogenesis [71, 72]. Regardless, the regulatory pathway for *KMT2A* and *NPM1*‐mutated AML subtypes is more clearly defined and is probably why commercial development efforts have been focused on these patient cohorts. However, there is a broader spectrum of patients (e.g. those with *NUP98* or *NUP214* rearrangements, *UBTF* tandem duplications and other *HOX*‐mediated leukaemias) who could benefit from menin inhibition as the field evolves, due to the dependency of their leukaemic cells on the menin‐KMT2A interaction.

Menin inhibitors are currently undergoing investigation as monotherapies or in combination with intensive chemotherapies (typically for younger, fit patients) and more targeted treatment regimens (for older and/or unfit patients). Clinical exploration has centred on menin inhibition within the R/R AML setting (Table 2), and this is further underscored by the FDA's approval of revumenib for KMT2Ar R/R AML in patients aged 1 year or older [48]. However, there is growing interest in expanding into frontline regimens for newly diagnosed AML and potentially utilising menin inhibitors as maintenance therapy, even after allogeneic stem cell transplantation, where appropriate. Additionally, the effectiveness of menin inhibitors in paediatric patients is also being explored via the extension of adult study criteria to include younger individuals in clinical trials (bolded trials in Table 2). This will hopefully accelerate the establishment of safe and effective paediatric dosages, potentially in combination with appropriate multi‐agent chemotherapy. Collectively, the data from early‐stage clinical trials is promising and demonstrates proof‐of‐concept; however, it is clear that larger, randomised Phase III studies are necessary for validation. Furthermore, there remains a lack of long‐term data on the durability of clinical responses, the potential for late‐emerging toxicities and overall survival benefits [73].

## Outlook and future considerations

The expansion of targeted treatment options for AML offers improved efficacy and fewer toxicities, culminating in a greater quality of life for patients. Menin inhibition will undoubtedly reshape the treatment paradigm for patients with *KMT2A*r and other menin‐sensitive genetic alterations that were beyond the scope of this review, including: *NPM1* mutations, *NUP98* and *NUP214* rearrangements, and *UBTF* tandem duplications. To help achieve this, the creation of a predictive/pharmacodynamic biomarker assay that is capable of reliably detecting *HOX* gene expression (or a characteristic gene signature based on menin‐KMT2A binding motifs) would be ideal for: (a) identifying and expanding into a larger subset of patients that may benefit from menin inhibition; (b) predicting the responses of a patient to different menin inhibitors *ex vivo* to better inform and tailor treatment regimens; and (c) helping to monitor treatment responses (which should also be supplemented with standardised prospective monitoring of *MEN1* mutation status). Furthermore, while the emergence of drug resistance is inevitable, ongoing research into combining menin inhibitors with other targeted therapies that could prevent and/or overcome acquired resistance is encouraging. However, realising the full potential of menin inhibition within the paediatric/childhood setting of AML will require global co‐operation. Securing ethical approval for trial initiation, overcoming patient recruitment and enrolment hurdles, sharing data and resources, as well as forging collaborative partnerships between industry, academic institutions, government agencies and clinicians are all vital components for accelerating progress and ultimately ensuring broad access to this promising therapy for all children and adolescents with KMT2Ar AML.

## Conflicts of interest

LER and KML have no conflicts of interest to declare. GG received an honorarium from Novartis, GSK. GG also reports financial support in the form of an Education Support/Travel award from Novartis.

## Author contributions

Drafting of the manuscript and articulation of the primary content were undertaken by LER. Revisions to enhance the intellectual content and ensure accuracy were performed by LER, GG and KML. All authors have reviewed and approved the manuscript in its final form.
